# Validation of International Cognitive Ability Resource (ICAR) Implemented in Mobile Toolbox (MTB)

**DOI:** 10.3390/jintelligence13120154

**Published:** 2025-12-01

**Authors:** Stephanie Ruth Young, Jiwon Kim, Kiley McKee, Danielle Rothschild Doyle, Miriam A. Novack, William Revelle, Richard Gershon, Elizabeth M. Dworak

**Affiliations:** 1 Department of Medical Social Sciences, Northwestern University Feinberg School of Medicine, Chicago, IL 60611, USA; 2 Department of Psychology, Northwestern University Weinberg College of Arts and Sciences, Evanston, IL 60208, USA

**Keywords:** International Cognitive Ability Resources (ICAR), Mobile Toolbox (MTB)

## Abstract

Standardized cognitive assessments are essential in research but often limited by proprietary restrictions and methodological constraints. This study evaluates the psychometric properties of two public-domain International Cognitive Ability Resource (ICAR) measures implemented in the Mobile Toolbox (MTB) assessment library: Puzzle Completion and Block Rotation. Using a sample of 100 adults (18–82 years), we assessed internal consistency, test–retest reliability, and construct validity compared to gold-standard measures. Results demonstrated acceptable reliability for both Puzzle Completion and Block Rotation. Each measure showed moderate to strong correlations with respective gold-standard assessments: Puzzle Completion correlated with Raven’s Progressive Matrices (r = 0.40), and Block Rotation with Mental Rotation Test (r = 0.46). Practice effects were non-significant. Both demonstrated the ability to discriminate between verbal and nonverbal abilities. Findings were consistent with previous ICAR validations, suggesting MTB provides a viable option for remote self-administration while preserving measurement integrity. This enables larger sample collection and ecological assessment of cognitive abilities outside of laboratory settings.

## 1. Introduction

Cognitive assessments play a critical role across research fields, informing our understanding of cognitive development, individual differences, clinical outcomes, and personal well-being. However, access to high-quality, validated cognitive measures is often limited by proprietary restrictions, high costs, or methodological constraints. The International Cognitive Ability Resource (ICAR) was developed to address these challenges by offering a well-validated nonproprietary cognitive assessment battery freely available to researchers (Condon and Revelle 2014). Since its introduction a decade ago, ICAR has been used in over 200 research studies and counting (Dworak et al. 2021). The ICAR is an open-source, public-domain tool that allows researchers to submit, use, and validate cognitive tests. Developed by an international collaboration of cognitive ability testing researchers, it was initially conceptualized as a broad library of public-domain, self-administered cognitive tests for use on mobile smartphones and the web. Its goals have grown, and it now accepts contributions from external groups, enhancing open science scholarship and supporting the sharing of assessment materials. The resource offers over 20 measures and continues to grow, though the original four ICAR measures: Three-Dimensional Rotation, Matrix Reasoning, Letter Number Series, and Verbal Reasoning as well as the 16-item sample test tend to be the most used in research (Dworak et al. 2021).

The ICAR measures have been primarily administered online, such as through the Synthetic Aperture Personality Assessment (Revelle et al. 2016), or using a paper format (Young and Keith 2020). As digital research methodologies evolve, there is growing demand for cognitive assessments that can be flexibly administered across platforms, particularly on mobile devices. Given smartphones’ ubiquity and their potential to enable remote, scalable cognitive testing, adapting ICAR measures for standardized offline mobile administration represents a crucial advancement in expanding accessibility and participation in cognitive research. This is especially important as ICAR had already been designed for smartphone administration via a web browser. Yet without a thorough evaluation of its psychometric properties when administered via smartphones, researchers will struggle to interpret results and compare findings across data collection approaches. With accompanying validity and reliability evidence, smartphone-administered ICAR measures would provide researchers with a trusted option for remote cognitive testing and enable high-frequency research designs, such as ecological momentary assessment, experience sampling methods, or daily diaries.

The NIH-funded Mobile Toolbox (Gershon et al. 2022) provides an ideal framework for integrating ICAR measures into mobile research methodologies. MTB provides an assessment library that researchers can use, leveraging the well-established research platform REDCap (Harris et al. 2009, 2019) and the MyCap Mobile App (Harris et al. 2022). This enables researchers to create assessment batteries that participants can complete remotely on their smartphones. These assessments are designed to be fully functional offline or with limited internet connectivity, ensuring flexibility and accessibility for participants in areas with unreliable or no internet access. This format enhances the usability of ICAR measures, which were not previously optimized for offline mobile assessment or tailored administration. Using MTB versions of the ICAR measures allows researchers to simultaneously collect data on multiple constructs, selecting from both the MTB library of performance-based neurocognitive measures and thousands of existing survey measures in the REDCap library (Obeid et al. 2013). The REDCap system’s MyCap Mobile App offers advanced features, such as participant reminders or notifications, messaging, and the capacity for both cross-sectional and longitudinal designs. These features in conjunction with remote assessment capabilities reduce the burden on both researchers and participants while facilitating streamlined data management and participant tracking.

To leverage MTB ICAR measures effectively, we must establish that they maintain psychometric integrity in their mobile format. Therefore, this study evaluates the psychometric properties of two core ICAR measures—Progressive Matrices (PM) and Three-Dimensional Rotation (R3D)—as implemented within MTB. This study builds upon the work of other external validations, especially comparisons of the ICAR with the WAIS-IV (Young and Keith 2020).

Given theoretical and empirical evidence that fluid reasoning (Gf) and visual–spatial processing (Gv) are interrelated (Carroll 1993; Schneider and McGrew 2012), as well as the overlap seen in previous validations (Condon and Revelle 2014; Young and Keith 2020), we expected the two ICAR measures to show moderate cross-domain associations. Therefore, convergent and discriminant validity were anticipated to manifest not as strict separation between constructs, but rather as *relative* differences in the strength of correlations—with each measure showing its strongest association with its respective gold-standard task and weaker, though nonzero, relations with measures of other domains.

Specifically, we address three research questions:

RQ1 (Reliability): Do the MTB ICAR measures demonstrate acceptable internal consistency and test–retest reliability?
RQ2 (Construct Validity): Do the MTB ICAR measures show expected convergent validity with established “gold-standard” assessments of fluid reasoning and visual–spatial ability, and discriminant validity with a measure of verbal comprehension?
RQ3 (Comparison to Existing Evidence): How do the psychometric properties of the MTB ICAR measures compare with those reported in prior ICAR validation studies using web- or lab-based administration formats?

By addressing these questions, we provide critical insights into the feasibility of mobile-based ICAR assessments and their potential to enhance cognitive research across diverse populations and settings. We hypothesized that each MTB ICAR measure would demonstrate moderate to strong correlations (r ≥ 0.40) with its corresponding gold-standard assessment, and smaller but non-zero correlations with measures of other domains, consistent with theoretical overlap between Gf and Gv abilities (Carroll 1993; Schneider and McGrew 2012).

## 2. Materials and Methods

All study methods and procedures were approved by the Northwestern University Institutional Review Board (STU00218146). All participants provided informed consent and were compensated for participation in the study.

### 2.1. Sample

A sample of 100 U.S.-based adults between the ages of 18 to 82 years old (*M* = 45.31 years, *SD* = 18.06 years) was recruited by a large market research firm through their database of research volunteers (see Table 1).

### 2.2. Measures

#### 2.2.1. ICAR Measures

The names of the ICAR measures were changed to be more participant-friendly and consistent with the rest of the MTB assessment library. The original versions of the measures have all demonstrated evidence of reliability and validity in independent validation studies (Condon and Revelle 2014; Jankovski et al. 2017; Young and Keith 2020). The MTB adaptations of ICAR measures involved optimizing item presentation for smaller smartphone screens, implementing touch-based response selection, and incorporating automatic data capture while maintaining the core cognitive demands of each task. Additionally, a set of practice items, demonstration items, and standardized instructions were added to the MTB versions of these measures. Further work was done to make a subset of the measures suitable for tailored administration using a computer adaptive test (CAT). Finally, two response options included in the original ICAR versions (“None of the above” and “I don’t know,”) were removed for the MTB adaptations given low endorsement rates in the extant SAPA data.

**Figure 1:**
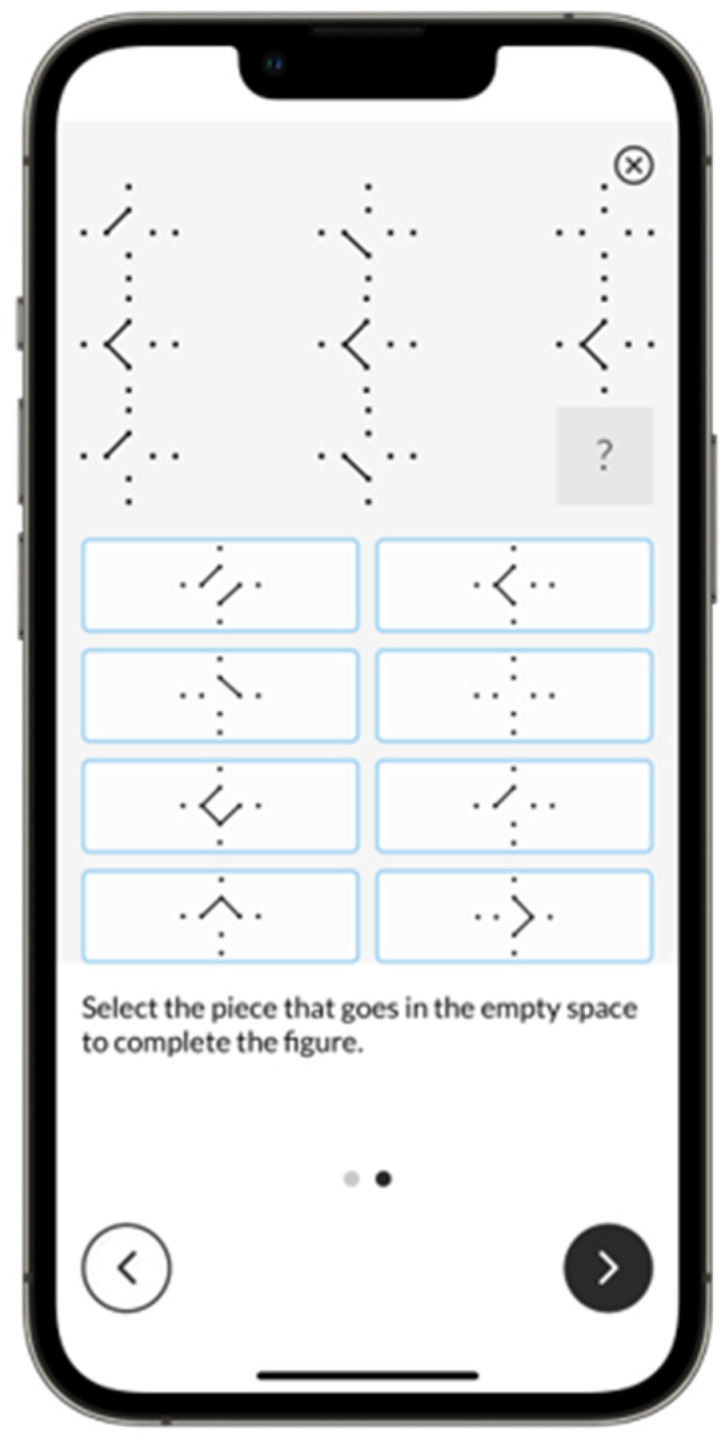
Example Puzzle Completion Screen.

*Puzzle Completion.* Puzzle Completion, also known as Progressive Matrices within ICAR, is modeled after Raven’s Progressive Matrices and assesses fluid or nonverbal intelligence. Participants are asked to select the missing piece in a three-by-three matrix from eight response options (Figure 1). The MTB version used a fixed form with 30 of the original items. A two-parameter logistic (2PL) item response theory (IRT) model was used for scoring.

**Figure 2:**
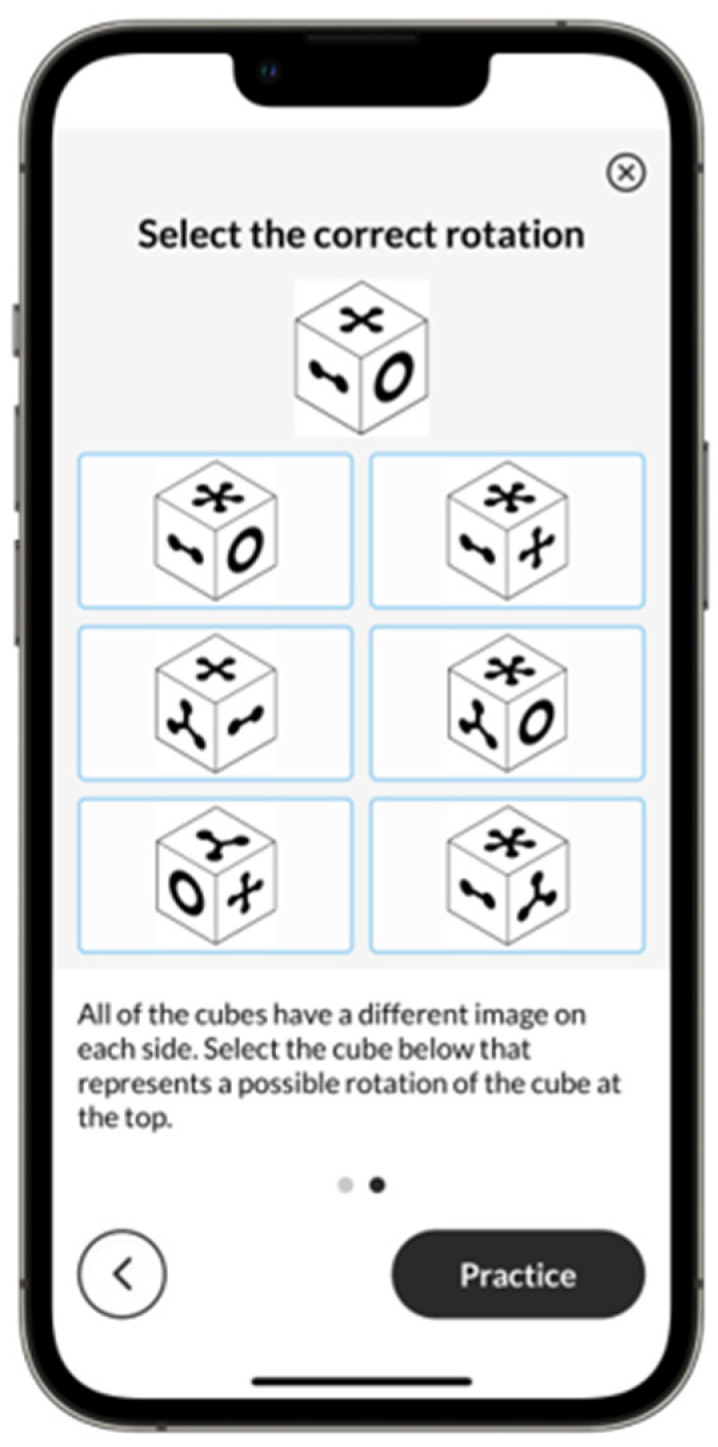
Example Block Rotation Screen.

*Block Rotation.* Block Rotation, also known as Three-Dimensional Rotation within ICAR, is modeled after Gittler and Glück’s (1998) items and assesses visual-spatial rotation abilities. Participants are asked to select a possible rotation of a target cube from six response options (Figure 2). The MTB version uses a CAT version with a maximum of 25 items, scored using a 2PL IRT model. The stopping rule was based on the standard error of 0.5, with a maximum of 25 items.

#### 2.2.2. External Gold Standard Measures

A battery of well-validated and established external “gold standard” measures was administered to participants to assess the convergent and divergent construct validity of the measures under investigation. These external measures provided a benchmark for comparing the measurements of the intended constructs. As evidence of convergent and discriminant validity, we expected measures of similar constructs to correlate highly and measures of dissimilar constructs to demonstrate smaller correlations.

*Raven’s Progressive Matrices (RPM; Self-Administered Tablet Format).* Raven’s Progressive Matrices is an assessment of nonverbal reasoning and fluid intelligence. The test consists of visual patterns in which participants must identify the missing piece from a set of options to complete a logical sequence. RPM demonstrates evidence of reliability and validity (Cockcroft and Israel 2011) and serves as a point of convergent validity for Puzzle Completion due to its similar matrix-based format and its status as the original model for this type of nonverbal reasoning assessment.

*Mental Rotation Test (MRT; Self-Administered; Paper Format).* The MRT is a widely used measure of spatial visualization ability, assessing capacity to mentally manipulate three-dimensional objects (Peters et al. 1995; Vandenberg and Kuse 1978). The MRT is based on Shepard and Metzler’s original mental rotation test (Shepard and Metzler 1971), which presented participants with three-dimensional cube figures. Participants had to mentally manipulate and rotate these figures in order to compare if they were the same figure or mirror reflections of each other. The MRT uses the same kind of cube figures and comparison. Each item consists of a target figure along with two correct and two incorrect rotation response options. The items have been used extensively in visual-spatial research and are associated with activity in visual-spatial processing areas of the brain on fMRI studies (Cohen et al. 1996). The MRT serves as a point of convergent validity for Block Rotation because it is the most established measure of three-dimensional mental rotation ability in cognitive science literature. The MRT is self-administered with a proctor and is typically timed, though we examined untimed scores for consistency with the ICAR measures.

*Visualization—Block Rotation (V-BR; Examiner Administered; Paper Format).* A subtest from the Woodcock Johnson, Fourth Edition (WJ-IV), Visualization is an examiner administered task that assesses spatial and visual processing skills. Specifically, we used the Visualization sub-scale Block Rotation, which focuses on mental manipulation and rotation of objects. It is frequently used in educational and clinical settings to evaluate cognitive strengths and weaknesses in spatial processing as part of a broader assessment of intellectual functioning (Schrank et al. 2014). Visualization serves as an additional point of convergent validity for Block Rotation beyond the MRT because it provides a well-standardized clinical measure of spatial processing in an untimed, examiner administered format.

*Peabody Picture Vocabulary Test, Fifth Edition (PPVT-5; Self-Administered Tablet Format).* The PPVT-5 is a standardized assessment of receptive vocabulary knowledge. In this test, examinees are shown sets of four pictures and asked to select the picture that best represents a spoken word. The PPVT-5 demonstrates strong psychometric properties including excellent internal consistency (α > 0.90) and test–retest reliability (*r* > 0.90) (Dunn and Dunn 1965). The PPVT-5 serves as a point of divergent validity for both measures.

### 2.3. Procedure

Participants were invited to the lab for their baseline visit where they were guided through downloading the MyCap Mobile App onto their personal smartphones (Android or iPhone). Order of administration was randomized, with half the sample self-administering the ICAR measures first, and the other half being administered the external assessments first. The order of administration was counterbalanced across participants to control for potential order or fatigue effects.

Trained examiners followed standardized administration procedures for all external measures, except for MRT, which we administered untimed for consistency with the other measures. Within 11 to 17 days later (*M* = 12.11; *SD* = 1.65), a subsample of participants (*n* = 56) completed the ICAR measures again remotely on their own smartphones in order to assess test–retest reliability.

### 2.4. Analysis

All analyses were conducted using R statistical software version 4.5.0 (R Core Team 2025). Gold-standard measures were scored according to their standardized protocols, with raw scores converted to standard scores where appropriate for comparison. The score distributions of each MTB ICAR measure’s item set were evaluated for floor and ceiling effects. There were two items on the Block Rotation measure in which no participants responded correctly, and they were thus permanently from the item set and not included in analyses. Given the high number and difficulty of the Puzzle Completion and Block Rotation items, participants who spent less than 2 min total on the subtest (Puzzle Completion: *n* = 3; Block Rotation: *n* = 7) were considered outliers indicative of poor effort and dropped from the sample. As there is no time limit on the self-administered ICAR tests, we examined the mean completion time.

Internal consistency was evaluated using three complementary indices—(1) empirical reliability of IRT-based theta scores, (2) Cronbach’s α, and (3) McDonald’s Total ω_t_—to provide a comprehensive assessment of measurement precision. Empirical reliability represents the IRT analogue of α, estimating the proportion of observed-score variance attributable to true-score variance across the latent trait continuum, while Cronbach’s α is a classical test theory index that assumes tau-equivalence (equal factor loadings) and reflects the degree to which items measure a common construct. McDonald’s Total ω_t_ relaxes the tau-equivalence assumption and provides a more accurate estimate when items have differing loadings. For all three metrics, values ≥ 0.70 were interpreted as acceptable, ≥0.80 as good, and ≥0.90 as excellent internal consistency (Nunnally and Bernstein 1994). We also examined the average inter-item correlation ($\overset{-}{r}$) to assess item homogeneity and potential redundancy. Mean inter-item correlations in the range of 0.15–0.50 were considered desirable: values below 0.15 suggest heterogeneous content coverage, whereas values above 0.50 may indicate item redundancy or overly narrow construct sampling (Clark and Watson 1995).

Test–retest reliability was examined through Pearson correlations between the baseline and follow up administrations, and intraclass correlations (ICC). We also examined practice effects through the magnitude of mean differences between baseline and retest scores via Cohen’s d.

The convergent validity of each MTB ICAR measure was assessed through Pearson correlations (pairwise deletion) with external gold standard counterparts. As these measures do not have a verbal component, discriminant validity was examined through Pearson correlations with the PPVT-5. As an additional point of validity, correlations with age were examined with the expectation that, based on prior research, scores would decrease with increasing age. We used standard guidelines to judge the magnitude of effect sizes across analyses (e.g., Cohen’s d) (Cohen 2013).

Given our final analytic sample sizes (approximately 90–100 participants per correlation), the study had 80% power at α = 0.05 (two-tailed) to detect moderate correlations (*r* ≥ 0.28), indicating adequate sensitivity to identify convergence correlations but limited precision to distinguish small differences between correlations.

## 3. Results

Internal consistency estimates indicated strong reliability for both MTB ICAR measures (Table 2). Internal consistency across all indices—empirical reliability, Cronbach’s α, and McDonald’s Total ω_t_—indicated that both MTB ICAR measures demonstrated good to excellent reliability. The average inter-item correlations fell within the recommended 0.15–0.50 range, suggesting cohesive item sets that capture a common construct without redundancy. Test–retest correlations and ICCs showed strong temporal stability for Puzzle Completion and moderate stability for Block Rotation, consistent with the expected variability in self-administered cognitive tasks.

Practice effects were non-significant for both measures (Puzzle Completion *t*(61) = 0.004; *p* = 0.997; Block Rotation t(55) = 0.139, *p* = 0.171), with a negligible effect size for Puzzle Completion and small effect size for Block Rotation.

Both MTB ICAR measures demonstrated the expected convergent and discriminant validity patterns (Table 3). Puzzle Completion correlated most strongly with Raven’s Progressive Matrices, supporting its validity as a measure of nonverbal reasoning. Moderate correlations were also observed with Mental Rotation and Visualization–Block Rotation, consistent with theoretical overlap between reasoning and visuospatial processing domains. Block Rotation showed robust convergence with both spatial reference measures—Mental Rotation and Visualization–Block Rotation—and a moderate association with Raven’s, suggesting shared variance between spatial and reasoning constructs. External measures (i.e., Mental Rotation, Visualization Block Rotation, PPVT-5, Raven’s Progressive Matrices) tended to show relatively larger correlations with each other than with the ICAR measures but in a similar pattern (lower correlations between verbal and nonverbal measures).

Discriminant validity was supported by weaker or nonsignificant correlations with the verbal measure PPVT-5, indicating that the ICAR measures assess nonverbal ability rather than general language knowledge. Both ICAR measures demonstrated expected negative correlations with age, though this effect was statistically significant only for Block Rotation.

## 4. Discussion

This study evaluated the psychometric properties of ICAR measures implemented as self-administered smartphone tests in the MTB: Puzzle Completion and Block Rotation. Our results address questions regarding the reliability and construct validity of the measures and compare these findings with previous ICAR studies. Findings support the use of the MTB ICAR measures as psychometrically sound assessments of nonverbal reasoning.

*Reliability of MTB ICAR Measures (RQ1).* Our findings demonstrate strong internal consistency for both measures, supported by converging evidence from empirical reliability, Cronbach’s α, and McDonald’s Total ω_t_. All coefficients exceeded recommended benchmarks (≥0.70), indicating that both tasks yield precise and internally consistent estimates of nonverbal ability. Average inter-item correlations fell within the ideal range (0.15–0.50), suggesting balanced item homogeneity without redundancy. Test–retest analyses further indicated strong temporal stability for Puzzle Completion and moderate stability for Block Rotation, consistent with expected variability in self-administered tasks. No significant practice effects were observed, likely reflecting the high difficulty of the tasks. Although fluid reasoning tasks can show retest gains (Kail 1986; Sánchez-Benavides et al. 2016), task difficulty can mitigate improvement from repeated exposure (Basso et al. 1999; Fehringer 2023). Overall, the MTB ICAR measures demonstrate excellent reliability and measurement precision across both classical and IRT frameworks.

*Construct Validity Compared to a Gold-Standard Assessments (RQ2).* Each MTB ICAR measure showed varying degrees of evidence of convergent validity with its corresponding gold-standard assessment. The consistent pattern of correlations across our mobile administration and previous web-based validations suggests the cognitive constructs measured by ICAR are robust across diverse platforms, supporting the use of these measures in various research contexts regardless of administration format. Puzzle Completion correlated most strongly with Raven’s Progressive Matrices, supporting its validity as a measure of nonverbal reasoning. Moderate correlations with Mental Rotation and Visualization–Block Rotation further suggest partial overlap with visuospatial processing. This pattern of correlations supports the construct validity of Puzzle Completion as a measure of nonverbal reasoning while acknowledging its multifaceted nature.

Block Rotation showed strong convergence with both spatial reference measures—Mental Rotation and Visualization–Block Rotation—and a moderate association with Raven’s, reflecting shared variance between spatial and reasoning processes. The ICAR Block Rotation test uses singular cubes with different symbols on each face, unlike the MRT and V-BR which use three-dimensional figures with multiple stacked cubes. This stimuli difference may make ICAR Block Rotation more susceptible to non-rotation strategies; individuals may rely more on analytic strategies comparing the symbols rather than mental rotation (Boone and Hegarty 2017; Hegarty 2018). While our results show a strong correlation between Block Rotation and the two cube figure mental rotation tests, the correlation is not as strong as between the two gold-standard measures themselves, suggesting Block Rotation may be less reliant specifically on holistic rotation strategies than more general spatial ability.

Discriminant validity was supported by weak or nonsignificant correlations with the PPVT-5. This pattern indicates that both ICAR measures assess nonverbal abilities as intended. Moreover, the low correlations underscore that the ICAR measures are specific to reasoning and visuospatial processes rather than a gross reflection of overall cognitive ability indexed by the PPVT-5. It is worth noting that correlations between the external gold-standard measures were relatively higher among each other than with the ICAR measures, which is likely due to shared method variance. The external gold-standard measures were all examiner-administered in person, and tasks sharing the same administration format tend to show higher intercorrelations because of common method factors (e.g., examiner interaction, standardized testing environments). In contrast, the self-administered mobile format of the ICAR measures introduces different contextual influences, reducing shared method variance with the gold-standard tasks but not necessarily indicating lower validity.

Negative correlations with age were observed for both measures, reaching significance only for Block Rotation, consistent with established evidence of age-related decline in visuospatial processing. (Salthouse 2019), providing additional evidence of construct validity. Our sample’s wide age range (18–82 years) revealed expected negative correlations with age across both measures consistent with established literature on fluid cognitive abilities, but stronger than relationships observed in Young’s primarily college-aged sample. We could not find other ICAR studies that directly reported age correlations, but at least one study have demonstrated the ICAR’s validity in older adults (Young and Keith 2020).

*Comparison to Existing Literature (RQ3).* Our findings largely parallel Young’s (2020) WAIS-IV validation results, which found the strongest associations with measures of fluid and visuospatial reasoning. It is also important to note that Young (2020) used the original Matrix Reasoning task from the ICAR, while we used Progressive Matrices task, which is a similar paradigm with different item content.

Prior studies have questioned whether matrix reasoning tasks exclusively measure fluid intelligence or also incorporate visual–spatial abilities (Waschl et al. 2017). Our results support this multifaceted interpretation: Puzzle Completion was most strongly associated with measures of fluid reasoning but also showed moderate relationships with visuospatial measures, indicating shared cognitive demands across domains. Additionally, Raven’s Matrices comprises visual analogy problems (Carpenter et al. 1990), which has been found to be highly related to mental rotation ability and block building tasks (Carpenter et al. 1990; Mackintosh and Bennett 2005). Our findings suggest Puzzle Completion and Raven’s Matrices require similar cognitive demands, as we see expected correlations between Puzzle Completion and Raven’s Matrices, as well as with the specific visuo-spatial measures. Similarly, the correlation pattern we found for Block Rotation aligns with previous findings comparing ICAR domains with the WAIS-IV (Young and Keith 2020) where spatial reasoning tasks engage multiple cognitive processes beyond visual–spatial abilities.

Overall, these findings demonstrate that the MTB implementations of the ICAR measures maintain their convergent validity with external gold standard “nonverbal” ability measures, primarily fluid and visual reasoning tasks, but are complex and multifaceted rather than pure measures of single abilities. The pattern of correlations across measures suggests there is considerable overlap in the cognitive processes engaged during performance, and much of the variance may be attributable to the influence of overall intelligence.

*Limitations.* Several limitations should be considered when interpreting these findings. Although the analytic sample was powered to detect moderate correlations between convergent measures, it was not powered to detect small differences between correlations. While the observed correlations were moderate, this pattern is consistent with theoretical expectations that fluid reasoning (Gf) and visual–spatial processing (Gv) reflect related but partially distinct constructs (Carroll 1993; Schneider and McGrew 2012), indicating that the ICAR measures capture both shared cognitive processes and task-specific demands. Another consideration is that the sample was intentionally heterogeneous in age and educational background to enhance generalizability and representation, but this variability may have inflated variance and covariance estimates, potentially influencing the strength of observed relationships.

In addition, our inferences depend on the validity of the external “gold-standard” measures used for comparison. Some of these measures—particularly the Vandenberg and Kuse Mental Rotation Test—have themselves been critiqued for limited construct coverage within the broader domain of spatial cognition (Uttal et al. 2024). Finally, as the MTB ICAR measures were self-administered on participants’ personal smartphones, greater response variability was expected relative to examiner-administered tests. Prior work has shown that self-administered cognitive assessments can yield broader score distributions and somewhat weaker correspondence with laboratory measures (Simmons et al. 2023). Despite these limitations, the MTB ICAR measures still demonstrated meaningful convergent validity with examiner-administered tasks, supporting their utility for scalable, remote cognitive testing.

Finally, given the self-administration of MTB, we anticipate a certain level of noise in our data that would not be expected from experimenter-administered measures. Recent research has shown that the relationship between self-administered and in-lab-administered performance-based cognitive measures can be weak, with poorer performance and a larger range of scores on the self-administered version (Simmons et al. 2023). Furthermore, since MTB is administered on a smartphone, differences in individual experience with operating smartphones might also affect performance on the MTB ICAR measures. However, in this study, the gold-standard measures were not self-administered, and the MTB ICAR measures still demonstrated convergent validity with these gold-standard measures.

## 5. Conclusions

In this study, we examined the psychometric properties and validity of the ICAR measures of Puzzle Completion and Block Rotation when self-administered via MTB. The MTB versions of Block Rotation and Puzzle Completion are valid measures of nonverbal reasoning, likely measuring both visual and nonvisual processes of fluid intelligence. Overall, the findings were consistent with previous validations of the ICAR, indicating that the adaptation of measures had minimal impact on their psychometric properties. This confirms that MTB is a viable platform for remote self-administration of these measures. MTB opens new possibilities for large-scale, remote cognitive assessment while preserving measurement integrity and construct validity of the ICAR measures. The ICAR measures are well suited for many areas of research, including spatial abilities (Farran et al. 2024; Lakin et al. 2024; Uttal et al. 2024), giftedness (Young et al. 2021) and personality and individual difference (Dworak et al. 2021). A variety of MTB measures spanning multiple cognitive domains are available to researchers for free via REDCap, and more information is available at www.mobiletoolbox.org. MTB contributes to the evolution of intelligence research methodology, enabling collection of larger and more diverse samples, longitudinal measurement, and ecological assessment of cognitive abilities in real-world contexts rather than laboratory settings.

## Figures and Tables

**Table 1:** Sample Descriptives (N).

Total Sample	100 (%)
Gender	
Female	54
Male	46
Age Group	
18–29 years old	30
30–44 years old	26
45–59 years old	6
60–74 years old	35
75+ years old	3
Operating System	
iPhone	54
Android	46
Race	
White	64
Black or African American	20
Asian	1
Multiracial	1
Other/Prefer Not to Say	14
Ethnicity	
Hispanic or Latino	42
Not Hispanic or Latino	58
Education	
Less than a High School	2
High School Diploma or GED	33
Some College or Trade School	16
2-Year College Degree	9
Bachelor’s Degree	26
Graduate Degree	14

**Table 2:** Reliability and Validity Statistics for MTB ICAR Tests.

	**Puzzle Completion**		**Block Rotation**	
	**Statistic**	**N**	**Statistic**	**N**
**Completion Time in Minutes**				
Mean [Min, Max]	9.95 [2.77, 23.85]	95	9.95 [2.17, 40.86]	91
**Reliability**				
Internal Consistency	0.88	95	0.81	91
Average Inter-item correlation	0.23	95	0.17	91
Cronbach’s α	0.90	95	0.93	91
McDonald’s Total ω_t_	0.90	95	0.89	91
Test–Retest	0.82	63	0.63	56
ICC [95% CI]	0.77 [0.64, 0.85]	63	0.60 [0.40, 0.74]	56
**Practice Effects**				
Mean Difference T2-T1 [95% CI]	0.001 [−0.309, 0.312]	62	0.409 [−0.182, 1.001]	56
Cohen’s d	0.000	62	0.204	56
**Age Correlation** [95% CI]	−0.154 [−0.35, 0.045 ]	95	−0.404 [−0.559, −0.212 ]	91

*Note.* Bolded numbers show the primary intended comparison. Mean Completion Time was reported in minutes. The initial analytic sample was 98 due to two cases with missing data. Three cases from Puzzle Completion and seven cases from Block Rotation were subsequently removed due to indications of careless responding (<2 min completion time).

**Table 3:** Correlation Table with Convergent and Discriminant Measures (N = 91).

	MTB ICAR		Convergent Measures			Discriminant Measure
	BR	PC	MRT	WJ-Viz	Raven’s	PPVT-5
BR	1					
PC	0.42 ***	1				
MRT	0.46 ***	0.36 ***	1			
WJ-Viz	0.51 ***	0.31 **	0.63 ***	1		
Raven’s	0.39 ***	0.39 ***	0.55 ***	0.60 ***	1	
PPVT-5	0.23 *	0.18	0.39 ***	0.31 **	0.44 ***	1

Note. Entries are Pearson’s *r* (two-tailed). BR = ICAR Block Rotation; PC = ICAR Puzzle Completion; MRT = Mental Rotation Test; WJ-Viz = Woodcock–Johnson IV Visualization (Block Rotation); PPVT-5 = Peabody Picture Vocabulary Test–5. * *p* < .05, ** *p* < .01, *** *p* < .001.

## Data Availability

The de-identified data presented in this study are available on request from the corresponding author to protect from potential violations of privacy in the small data set.

## Abbreviations

The following abbreviations are used in this manuscript:

ICAR	International Cognitive Ability Resource
MTB	Mobile Toolbox
SAPA	Synthetic Aperture Personality Assessment
RPM	Raven’s Progressive Matrices
MRT	Mental Rotation Test
WJ-IV	Woodcock Johnson, Fourth Edition
PPVT-5	Peabody Picture Vocabulary Test
2PL	Two parameter logistic
IRT	Item Response Theory
ICC	Intraclass Correlation

## References

[B1-jintelligence-13-00154] Basso Michael R., Bornstein Robert A., Lang Jennifer M. (1999). Practice Effects on Commonly Used Measures of Executive Function Across Twelve Months. The Clinical Neuropsychologist.

[B2-jintelligence-13-00154] Boone Alexander P., Hegarty Mary (2017). Sex differences in mental rotation tasks: Not just in the mental rotation process!. Journal of Experimental Psychology: Learning, Memory, and Cognition.

[B3-jintelligence-13-00154] Carpenter Patricia A., Just Marcel A., Shell Peter (1990). What one intelligence test measures: A theoretical account of the processing in the Raven Progressive Matrices Test. Psychological Review.

[B4-jintelligence-13-00154] Carroll John B. (1993). Human Cognitive Abilities: A Survey of Factor-Analytic Studies.

[B5-jintelligence-13-00154] Clark Lee Anna, Watson David (1995). Constructing validity: Basic issues in objective scale development. Psychological Assessment.

[B6-jintelligence-13-00154] Cockcroft Kate, Israel Nicky (2011). The Raven’s Advanced Progressive Matrices: A Comparison of Relationships with Verbal Ability Tests. South African Journal of Psychology.

[B7-jintelligence-13-00154] Cohen Jacob (2013). Statistical Power Analysis for the Behavioral Sciences.

[B8-jintelligence-13-00154] Cohen Mark S., Kosslyn Stephen M., Breiter Hans C., DiGirolamo Gregory J., Thompson William L., Anderson Adam K., Bookheimer Susan Y., Rosen Bruce R., Belliveau John W. (1996). Changes in cortical activity during mental rotation A mapping study using functional MRI. Brain.

[B9-jintelligence-13-00154] Condon David M., Revelle William (2014). The International Cognitive Ability Resource: Development and Initial Validation of a Public-Domain Measure. Intelligence.

[B10-jintelligence-13-00154] Dunn Lloyd M., Dunn Leota M. (1965). Peabody Picture Vocabulary Test–Third Edition. https://psycnet.apa.org/doiLanding?doi=10.1037/t15145-000.

[B11-jintelligence-13-00154] Dworak Elizabeth M., Revelle William, Doebler Philip, Condon David M. (2021). Using the International Cognitive Ability Resource as an open source tool to explore individual differences in cognitive ability. Personality and Individual Differences.

[B12-jintelligence-13-00154] Farran Emily K., McCarthy Sarah, Gilligan-Lee Katie A., Bates Kathryn E., Gripton Catherine (2024). Translating Research to Practice: Practitioner Use of the Spatial Reasoning Toolkit. Gifted Child Today.

[B13-jintelligence-13-00154] Fehringer Benedict C. O. F. (2023). Different Perspectives on Retest Effects in the Context of Spatial Thinking: Interplay of Behavioral Performance, Cognitive Processing, and Cognitive Workload. Journal of Intelligence.

[B14-jintelligence-13-00154] Gershon Richard C., Sliwinski Marti J., Mangravite Lara, King Jonathan W., Kaat Aaron J., Weiner Michael W., Rentz Dorene M. (2022). The Mobile Toolbox for monitoring cognitive function. The Lancet. Neurology.

[B15-jintelligence-13-00154] Gittler Georg, Glück Judith (1998). Differential transfer of learning: Effects of instruction in descriptive geometry on spatial test performance. Journal of Geometry and Graphics.

[B16-jintelligence-13-00154] Harris Paul A., Swafford Jonathan, Serdoz Emily S., Eidenmuller Jessica, Delacqua Giovanni, Jagtap Vaishali, Taylor Robert J., Gelbard Alexander, Cheng Alex C., Duda Stephany N. (2022). MyCap: A flexible and configurable platform for mobilizing the participant voice. JAMIA Open.

[B17-jintelligence-13-00154] Harris Paul A., Taylor Robert, Minor Brenda L., Elliott Veida, Fernandez Michelle, O’NEal Lindsay, McLeod Laura, Delacqua Giovanni, Delacqua Francesco, Kirby Jacqueline (2019). The REDCap consortium: Building an international community of software platform partners. Journal of Biomedical Informatics.

[B18-jintelligence-13-00154] Harris Paul A., Taylor Robert, Thielke Robert, Payne Jonathon, Gonzalez Nathaniel, Conde Jose G. (2009). Research electronic data capture (REDCap)—A metadata-driven methodology and workflow process for providing translational research informatics support. Journal of Biomedical Informatics.

[B19-jintelligence-13-00154] Hegarty Mary (2018). Ability and Sex Differences in Spatial Thinking: What Does the Mental Rotation Test Really Measure?. Psychonomic Bulletin & Review.

[B20-jintelligence-13-00154] Jankovski Jovana, Zecevic Ivana, Subotic Sinisa (2017). A preliminary examination of the ICAR Progressive Matrices test of intelligence. Empirical Studies in Psychology.

[B21-jintelligence-13-00154] Kail Robert (1986). The impact of extended practice on rate of mental rotation. Journal of Experimental Child Psychology.

[B22-jintelligence-13-00154] Lakin Joni M., Wai Jonathan, Olszewski-Kubilius Paula, Corwith Susan, Rothschild Danielle, Uttal David H. (2024). Spatial Thinking Across the Curriculum: Fruitfully Combining Research and Practice. Gifted Child Today.

[B23-jintelligence-13-00154] Mackintosh Nicholas J., Bennett Eric S. (2005). What do Raven’s Matrices measure? An analysis in terms of sex differences. Intelligence.

[B24-jintelligence-13-00154] Nunnally Jum C., Bernstein Ira H. (1994). Psychometric Theory.

[B25-jintelligence-13-00154] Obeid Jihad S., McGraw Catherine A., Minor Brenda L., Conde José G., Pawluk Robert, Lin Michael, Wang Janey, Banks Sean R., Hemphill Sheree A., Taylor Rob (2013). Procurement of shared data instruments for research electronic data capture (REDCap). Journal of Biomedical Informatics.

[B26-jintelligence-13-00154] Peters Michael, Laeng Bruno, Latham Kerry, Jackson Marla, Zaiyouna Raghad, Richardson Chris (1995). A redrawn Vandenberg and Kuse mental rotations test-different versions and factors that affect performance. Brain and Cognition.

[B27-jintelligence-13-00154] R Core Team (2025). *R: A Language and Environment for Statistical Computing*. Vienna: R Foundation for Statistical Computing. https://www.R-project.org/.

[B28-jintelligence-13-00154] Revelle William, Condon David M., Wilt Joshua, French Jason A., Brown Ashley, Elleman Lorien G. (2016). Web and phone based data collection using planned missing designs. Sage Handbook of Online Research Methods.

[B29-jintelligence-13-00154] Salthouse Timothy A. (2019). Trajectories of normal cognitive aging. Psychology and Aging.

[B30-jintelligence-13-00154] Sánchez-Benavides Gonzalo, Gispert Juan D., Fauria Karine, Molinuevo José Luis, Gramunt Nina (2016). Modeling practice effects in healthy middle-aged participants of the Alzheimer and Families parent cohort. Alzheimer’s & Dementia: Diagnosis, Assessment & Disease Monitoring.

[B31-jintelligence-13-00154] Schneider W. Joel, McGrew Kevin S. (2012). The Cattell-Horn-Carroll model of intelligence. Contemporary Intellectual Assessment: Theories, Tests, and Issues.

[B32-jintelligence-13-00154] Schrank Fredrick Allen, McGrew Kevin S., Mather Nancy, Wendling Barbara J., LaForte Erica M. (2014). Woodcock-Johnson IV Tests of Cognitive Abilities.

[B33-jintelligence-13-00154] Shepard Roger N., Metzler Jacqueline (1971). Mental Rotation of Three-Dimensional Objects. Science.

[B34-jintelligence-13-00154] Simmons Alexandra, McGatlin Kristen, Lustig Cindy (2023). How well do online, self-administered measures correspond to in-lab assessments? A preliminary examination of three measures in healthy older adults. Neuropsychology.

[B35-jintelligence-13-00154] Uttal David H., McKee Kiley, Simms Nina, Hegarty Mary, Newcombe Nora S. (2024). How can we best assess spatial skills? practical and conceptual challenges. Journal of Intelligence.

[B36-jintelligence-13-00154] Vandenberg Steven G., Kuse Allan R. (1978). Mental Rotations, a Group Test of Three-Dimensional Spatial Visualization. Perceptual and Motor Skills.

[B37-jintelligence-13-00154] Waschl Nicolette A., Nettelbeck Ted, Burns Nicholas R. (2017). The Role of Visuospatial Ability in the Raven’s Progressive Matrices. Journal of Individual Differences.

[B38-jintelligence-13-00154] Young Stephanie R., Keith Timothy Z. (2020). An examination of the convergent validity of the ICAR16 and WAIS-IV. Journal of Psychoeducational Assessment.

[B39-jintelligence-13-00154] Young Stephanie R., Maddocks Danika L. S., Carrigan Jamison E. (2021). The International Cognitive Ability Resource: A Free Cognitive Measure With Utility for Postsecondary Giftedness Research. Gifted Child Quarterly.

[B40-jintelligence-13-00154] Young Stephanie Ruth (2020). Valid Cognitive Ability Measures in the Public Domain: A Convergent Validity Study of the ICAR16 Using the WAIS-IV.

